# Neural Correlates of Non-clinical Internet Use in the Motivation Network and Its Modulation by Subclinical Autistic Traits

**DOI:** 10.3389/fnhum.2018.00493

**Published:** 2018-12-10

**Authors:** Hironobu Fujiwara, Sayaka Yoshimura, Kei Kobayashi, Tsukasa Ueno, Naoya Oishi, Toshiya Murai

**Affiliations:** ^1^Integrated Clinical Education Center, Kyoto University Hospital, Kyoto, Japan; ^2^Department of Neuropsychiatry, Graduate School of Medicine, Kyoto University, Kyoto, Japan; ^3^Artificial Intelligence Ethics and Society Team, RIKEN Center for Advanced Intelligence Project, Tokyo, Japan; ^4^Department of Neurodevelopmental Psychiatry, Habilitation and Rehabilitation, Kyoto University, Kyoto, Japan; ^5^Medical Innovation Center, Kyoto University Graduate School of Medicine, Kyoto, Japan

**Keywords:** internet use, autistic traits, motivation network, functional connectivity, mediation analysis

## Abstract

**Background:** Increasing evidence regarding the neural correlates of excessive or pathological internet use (IU) has accumulated in recent years, and comorbidity with depression and autism has been reported in multiple studies. However, psychological and neural correlates of non-clinical IU in healthy individuals remain unclear.

**Objectives:** The aim of the current study was to investigate the relationships between non-clinical IU and functional connectivity (FC), focusing on the brain’s motivation network. We sought to clarify the influence of depression and autistic traits on these relationships in healthy individuals.

**Methods:** Resting-state functional magnetic resonance imaging (fMRI) was performed in 119 healthy volunteers. IU, depression, and autistic traits were assessed using the Generalized Problematic Internet Use Scale 2 (GPIUS2), Beck Depression Inventory-II (BDI-II), and the autism spectrum quotient (AQ) scale, respectively. Correlational analyses were performed using CONN-software within the motivation-related network, which consisted of 22 brain regions defined by a previous response-conflict task-based fMRI study with a reward cue. We also performed mediation analyses via the bootstrap method.

**Results:** Total GPIUS2 scores were positively correlated with FC between the (a) left middle frontal gyrus (MFG) and bilateral medial prefrontal cortex; (b) left MFG and right supplementary motor area (SMA); (c) left MFG and right anterior insula, and (d) right MFG and right insula. The “Mood Regulation” subscale of the GPIUS2 was positively correlated with FC between left MFG and right SMA. The “Deficient Self-Regulation” subscale was positively correlated with FC between right MFG and right anterior insula (statistical thresholds, FDR < 0.05). Among these significant correlations, those between GPIUS2 (total and “Mood Regulation” subscale) scores and FC became stronger after controlling for AQ scores (total and “Attention Switching” subscale), indicating significant mediation by AQ (95% CI < 0.05). In contrast, BDI-II had no mediating effect.

**Conclusion:** Positive correlations between IU and FC in the motivation network may indicate health-promoting effects of non-clinical IU. However, this favorable association is attenuated in individuals with subclinical autistic traits, suggesting the importance of a personalized educational approach for these individuals in terms of adequate IU.

## Introduction

Internet use (IU) is a widespread and unique feature of modern human society. IU can involve various activities, such as obtaining information, gaming, online shopping, and communications via social networking services (SNS). Many aspects of daily life have been dramatically facilitated by IU.

A number of studies have suggested that excessive IU (EIU), particularly internet addiction and internet gaming disorder (American Psychiatric Association, 2013), have negative impacts on mental status (Chen et al., 2018). Among these two types of EIU, internet gaming disorders are particularly prevalent among younger populations, including adolescents, in East Asia (Liu J. et al., 2017).

Excessive IU is now regarded as a behavioral addiction that shares common pathophysiological mechanisms with compulsive shopping and pathological gambling. The reward-deficiency syndrome (RDS) hypothesis is the most influential predictive framework regarding the development of addiction, including behavioral addiction (Blum et al., 2008, 2014). The RDS hypothesis proposes that reduced dopaminergic neurotransmission, based on genetic background, underlies addiction (Blum et al., 2008).

Recent structural neuroimaging studies have demonstrated gray matter volume reduction in prefrontal brain regions including the orbitofrontal cortex (OFC) (Jin et al., 2016; Wang Z. et al., 2018), dorsolateral prefrontal cortex (DLPFC), anterior cingulate cortex (ACC), supplementary motor area (SMA) (Jin et al., 2016), and temporo-parietal regions (Wang Z. et al., 2018). A study has also reported decreased cortical thickness in the OFC and temporo-parietal regions (Wang Z. et al., 2018) in people with EIU. Additionally, diffusion tensor imaging has revealed lower structural connectivity in the ventral tegmental area (VTA)-nucleus accumbens (NAcc) tracts of people with EIU (Wang R. et al., 2018). These results indicate structural deficits in brain regions that are part of the reward system. These structural deficits have been demonstrated by different measures and imaging modalities, and are also consistent with positron emission tomography (PET) observations of decreased dopamine (DA) D_2_ receptor availability in the striatum of people with EIU, which indicates an attenuated reward system (Kim et al., 2011; Tian et al., 2014). A functional magnetic resonance imaging (fMRI) study also demonstrated the involvement of reward-system deficiency in people who frequently play online games, reporting lower brain activity in the ventral striatum when anticipating small or large monetary rewards (Hahn et al., 2014).

Resting-state fMRI (rs-fMRI) has revealed a number of networks that are consistently found in healthy people and represent specific patterns of synchronous activity (Rosazza and Minati, 2011). In addition to task-based fMRI, evaluation of resting-state functional connectivity (RSFC) provides an opportunity to characterize distributed circuit abnormalities in EIU (Ko et al., 2015; Wang et al., 2017). Jin et al. (2016) conducted a rs-fMRI analysis using brain regions in which volume reductions were found (OFC, DLPFC, ACC, and SMA) as the seeding areas and found that people with EIU exhibited lower functional connectivity (FC) between the seeds and several cortical regions, including temporal cortices, occipital cortices, and the insula (Ins). Moreover, they also found that EIU was associated with significantly lower FC between the seeds and subcortical regions, including the dorsal striatum, pallidum, and thalamus. Finally, some of these differences were associated with the severity of EIU (Jin et al., 2016). Another rs-fMRI study also demonstrated lower FC between VTA-NAcc and the OFC within reward circuitry (Wang R. et al., 2018) and lower RSFC between the amygdala and the DLPFC (Ko et al., 2015). Taken together, these findings from structural and functional imaging studies indicate that reward-system function is attenuated in EIU.

Key regions of the reward system constitute a functional network called the “motivation network” (Kinnison et al., 2012). This network includes brain regions activated by a reward-related paradigm (Padmala and Pessoa, 2011). It consists of 22 regions of interest (ROIs) that are mutually well-synchronized; the bilateral intraparietal sulcus (IPS), medial prefrontal cortex (MPFC), frontal eye field (FEF), middle frontal gyrus (MFG), anterior insula (aIns), midbrain (MB), putamen (Put), caudate (Caud), NAcc, left inferior parietal lobule (IPL), right rostral anterior cingulate cortex (rACC), SMA, and left precentral gyrus (PCG). In addition to midbrain and basal ganglia that constitute the central core of the reward system, these ROIs include several cortical regions that are well-synchronized with the core regions (Kinnison et al., 2012). Although the studies mentioned above have suggested abnormalities in multiple regions of the reward system in EIU, they have not explicitly investigated FC within the reward/motivation network.

At the same time, according to epidemiological surveys and their meta-analysis, EIU is associated with a variety of psychiatric disorders, typically depression, attention-deficit hyperactivity disorder, anxiety, obsessive-compulsive disorder, and autism spectrum disorder (ASD) (Ko et al., 2008; Carli et al., 2013; Ho et al., 2014; Nakayama et al., 2017; Kim et al., 2018; Kitazawa et al., 2018). Among these comorbidities, depression is the most commonly reported psychiatric feature. Kitazawa et al. (2018) demonstrated the relationship between EIU and depressive state in a general population of university students. Additionally, the short alleles of the serotonin transporter (5HTTLPR) polymorphism are known to be associated with depression (Wrase et al., 2006), and this common polymorphism was also demonstrated in EIU (Lee et al., 2008). Further, Kraut et al. (2002) introduced a “rich get richer” model in which the internet provides more benefits to those who are already well-adjusted, and in contrast, poorly adjusted adolescents with depression may suffer more deleterious effects from heavy IU, thereby creating a vicious circle.

Evidence also suggests a possible relationship between EIU and ASD (MacMullin et al., 2016; So et al., 2017), including subclinical autistic traits (Finkenauer et al., 2012; Romano et al., 2013, 2014; Liu S. et al., 2017). MacMullin et al. (2016) reported that individuals with ASD had greater compulsive IU than individuals without ASD. Finkenauer et al. (2012) reported that adults with high autistic traits could be accurately predicted to have compulsive IU. This finding is supported by several reports demonstrating positive correlations between IU and autistic traits in college students (Romano et al., 2013, 2014).

From a therapeutic point of view, treating psychiatric comorbidities of EIU is just as important as dealing with EIU itself. Nakayama et al. (2017) reported that psychosocial therapies (including cognitive behavioral therapy) for EIU and pharmacotherapies (including antidepressants) for comorbid psychiatric or developmental disorders have been effective at reducing the degree and symptoms of EIU. Likewise, Ko et al. (2008) suggested that psychiatric disorders comorbid with EIU should be evaluated and treated to prevent the worsening of EIU prognoses. They also proposed that recognizing the possibility of EIU is important when treating people with psychiatric disorders. In the current study, among the many possible comorbidities, we focused on depression (the most common comorbidity) and autistic traits that might be associated with restricted or repetitive/compulsive IU.

Despite the experimental evidence regarding EIU described above, the psychological correlates and neural underpinning of normal/adequate levels of IU have received relatively little research attention. Although the neural and psychological correlates of non-clinical IU are still unclear, we assumed two hypotheses: (1) the mechanisms of non-clinical IU are continuous with those of EIU, suggesting that IU, even at a mild level, constitutes a potential prodromal stage of EIU; and (2) the mechanisms of non-clinical IU are different from those of EIU, suggesting that, if not excessive, IU might have neutral effects or health benefits despite the potential for developing into a pathology. For example, a brain volumetric study demonstrated that online social network size was positively correlated with human brain size in healthy individuals (Kanai et al., 2012), indicating IU for social interaction might positively influence our brain. Furthermore, it is currently unclear how co-existent depressive tendencies or autistic traits affect the level of non-clinical IU, as well as the relationship between IU and brain function.

In the current study, we investigated the psychological correlates and neural underpinnings of normal levels of IU using resting-state fMRI (rs-fMRI), focusing on the FC within the reward/motivation network (Kinnison et al., 2012). We also investigated whether depression and autistic traits modulate the relationship between IU and FC. We hypothesized that the degree of IU would be associated with FC within the motivation network, and that depression and autistic traits would modulate the association between IU and FC.

## Materials and Methods

### Participants

Participants were 119 healthy volunteers (44 females; 35.7 ± 14.5 years, all right-handed). Two well-trained psychiatrists confirmed that no participant had any psychiatric disorder or severe medical or neurological illness.

Estimated intelligence quotients (IQs) were measured with the Japanese Version of the Adult Reading Test (JART; Matsuoka et al., 2006), and all participants fell within the normal range. After the experimental procedures were fully explained, all participants provided written informed consent before study participation.

The study was approved by Ethics Committee of the Kyoto University Graduate School and Faculty of Medicine, and was conducted in accordance with the guidelines of the Declaration of Helsinki.

### Psychological Questionnaire

#### Generalized Problematic Internet Use Scale 2 (GPIUS2; Caplan, 2010)

The GPIUS2 is a self-rating questionnaire that assesses an individual’s degree of IU. It contains 15 items, and responses are collected using an 8-point Likert-like scale. Total GPIUS2 scores range from 15 to 105, with five subscales: “Preference for Online Social Interaction (POSI),” “Mood Regulation (MR),” “Compulsive Use (CU),” “Cognitive Preoccupation (CP)” and “Negative Outcomes.” Each subscale has three items. An additional “Deficient Self-Regulation (DSR)” subscale can be calculated by summing CU and CP (Caplan, 2010). In the current study, the combined “Deficient Self-Regulation” subscale was used for statistical analyses. Higher GPIUS2 scores indicate greater IU. The original GPIUS2 has been validated (Caplan, 2010). We used the Japanese translation of the original version according to the following process: a qualified clinical psychiatrist together with a cognitive science researcher proficient in both Japanese and English translated the scale into Japanese. The psychiatrist had clinical experience in EIU-related cases, and the cognitive science researcher had experience in the translation and adaptation of psychological measures. When disagreements in language usage occurred, consensus was reached by involving a third researcher who was an expert in psychometrics. This helped ensure both linguistic and functional equivalence. The draft was then back-translated by two bilingual couples, in which at least one was a native Japanese speaker and at least one was a native English speaker. The psychiatrist and the cognitive science researcher compared the original scale to the back-translated version, and checked for semantic discrepancies. Finally, minor problems in Japanese expression were corrected.

Reliability and validity of the questionnaire was assessed as follows: First, the split-half coefficient scores (Spearman–Brown’s formula, ρ = 0.953) and internal consistency reliability (Cronbach’s α = 0.914) of the questionnaire suggested a high-level of reliability. Additionally, Cronbach’s alpha essentially did not change even after excluding any of the five subscales, indicating appropriate inclusion of all subscales in the original version. Furthermore, the mean GPIUS2 total score and the subscale scores in the current study (Table 1) were essentially the same as those from the study of healthy volunteers in which the original version of GPIUS2 was validated (Caplan, 2010). These results indicate that the Japanese translation of the GPIUS2 that we used in our study was constructed well in terms of reliability and concurrent validity.

**Table 1:** Participant demographics.

Variables		Mean (with *SD*)	
Age		35.7	(14.5)
Sex (male/ female)		74/45	
Education year		14.6	(2.4)
GPIUS2_total score		36.2	(15.7)
GPIUS2_preference for online communication		5.8	(2.7)
GPIUS2_mood regulation		9.0	(5.1)
GPIUS2_self regulation		15.0	(7.1)
GPIUS2_negative outcomes		6.4	(3.5)
BDI-II		6.1	(5.9)
AQ_total		18.5	(7.8)
AQ_social skill		3.6	(2.6)
AQ_attention switching		4.4	(2.2)
AQ_attention to details		4.0	(2.3)
AQ_communication		2.8	(2.2)
AQ_imagination		3.6	(2.1)


*GPIUS2, Generalized Problematic Internet Use Scale 2; BDI-II, Beck Depression Inventory-II; AQ, Autism Spectrum Quotient.*

#### Beck Depression Inventory-II (BDI-II; Beck et al., 1961)

The BDI-II was used as a measurement of depressive state. This scale consists of 21 items in which scores range from 0 to 63. Higher BDI-II scores indicate more severe depressive tendencies. We used the Japanese version of the BDI-II with validity that was previously confirmed by the Center for Epidemiologic Studies Depression Scale (CES-D), as well as confirmed internal consistency reliability (Cronbach’s α = 0.87) and item homogeneity (the mean inter-item correlation coefficient was 0.24) (Kojima et al., 2002).

#### Autism Spectrum Quotient (AQ)

The AQ is a self-report questionnaire for assessing the degree of autistic traits (Baron-Cohen et al., 2001; Wakabayashi et al., 2006). Each of the 50 items is given a score of one if the respondent reports abnormal or autistic-like behavior either mildly or strongly. In addition to the total score, the AQ generates scores for five subscales; “Social skill,” “Attention switching,” “Attention to detail,” “Communication,” and “Imagination.” Higher AQ scores indicate more severe autistic traits.

### MRI Acquisition

Structural MRI data were acquired using 3-dimensional magnetization-prepared rapid gradient-echo (3D-MPRAGE) sequences. Ten-minute resting-state data were acquired while participants kept their eyes open using a single-shot gradient-echo echo planar imaging (EPI) pulse sequence on a 3-Tesla MRI unit (Tim-Trio; Siemens, Erlangen, Germany) with a 40-mT/m gradient and a receiver-only 32-channel phased-array head coil. Head movement was minimized within the head coil with foam rubber pads. At resting-state data acquisition, we instructed participants to visually concentrate on a fixation cross in the center of the screen while avoiding thinking about anything specific. The parameters for the 3D-MPRAGE images were as follows: echo time (TE), 3.4 ms; repetition time (TR), 2000 ms; inversion time, 990 ms; field of view (FOV), 225 × 240 mm; matrix size, 240 × 256; resolution, 0.9375 mm × 0.9375 mm × 1.0 mm; and 208 total axial sections without intersection gaps. Parameters for the resting-state data were as follows: TE, 30 ms; TR, 2500 ms; flip angle, 80°; FOV, 212 × 212 mm; matrix size, 64 × 64; in-plane spatial resolution, 3.3125 mm × 3.3125 mm; 40 total axial slices; and slice thickness, 3.2 mm with 0.8-mm gaps in ascending order. A dual-echo gradient-echo dataset for B0-field mapping was also acquired for distortion correction.

### Image Preprocessing

The rs-fMRI dataset was corrected for EPI distortion using FMRIB’s Utility for Geometrically Unwarping EPIs (FUGUE), which is part of the FSL software package (FMRIB’s software library ver. 5.0.9)^1^ and which unwarps the EPI images based on fieldmap data. Artifact components and motion-related fluctuations were then removed from the images using FMRIB’s ICA-based X-noiseifier (FIX) (Griffanti et al., 2014).

The preprocessed rs-fMRI and structural MRI data were then processed using the CONN-fMRI Functional Connectivity toolbox (ver.17e)^2^ with the statistical parametric mapping software package SPM12 (Wellcome Trust Centre for Neuroimaging)^3^. First, all functional images were realigned and unwarped, slice-timing corrected, coregistered with structural data, spatially normalized into the standard MNI space (Montreal Neurological Institute, Canada), outlier detected (ART-based scrubbing), and smoothed using a Gaussian kernel with a full-width-at-half maximum (FWHM) of 8 mm. All preprocessing steps were conducted using a default preprocessing pipeline for volume-based analysis (to MNI-space). Structural data were segmented into gray matter, white matter (WM), and cerebrospinal fluid (CSF), and normalized in the same default preprocessing pipeline. Principal components of signals from WM and CSF, as well as translational and rotational movement parameters (with another six parameters representing their first-order temporal derivatives), were removed using covariate regression analysis by CONN. Using the implemented CompCor strategy (Behzadi et al., 2007), the effect of nuisance covariates, including fluctuations in rs-fMRI signals from WM, CSF, and their derivatives, as well as realignment parameter noise, were reduced. As recommended, band-pass filtering was performed with a frequency window of 0.008–0.09 Hz. This preprocessing step was found to increase retest reliability. Before running FIX, movement during rs-fMRI was evaluated using framewise displacement, which quantifies head motion between each volume of functional data (Power et al., 2012). Participants were excluded if the number of volumes in which head position was 0.5 mm different from adjacent volumes was more than 20%. In actuality, no participant was excluded according to this criterion.

### Functional Connectivity Analysis

We conducted a region of interest (ROI)-to-ROI FC analysis. We specified 22 spherical clusters with 10-mm diameters and peak-coordinates based on a previous motivation-related functional MRI study (Kinnison et al., 2012). The ROIs were located in the bilateral IPS (IPS_R: *x* = 24, *y* = -54, *z* = 40, IPS_L: -27, -52, 41), MPFC (MPFC_R: 6, 8, 39, MPFC_L: -8, 7, 39), FEF (FEF_R: 34, -11, 48, FEF_L: -31, -12, 50), MFG (MFG_R: 26, 46, 25, MFG_L: -28, 35, 29), aIns (aIns_R: 31, 17, 11, aIns_L: -35, 26, 5), Midbrain (MB_R: 7, -15, -8, MB_L: -10, -18, -8), Put (Put_R: 17, 9, -2, Put_L: -19, 9, 2), Caud (Caud_R: 10, 9, 2, Caud_L: -10, 9, 2), NAcc (NAcc_R: 13, 6, -7, NAcc_L: -13, 6, -7), left IPL (IPL_L: -28, -42, 41), right rACC (rACC_R: 13, 39, 8), right SMA (SMA_R: 0, -6, 57), and PCG (PCG_L: -48, -4, 37).

### Statistical Analysis

The association between GPIUS2 total score and each of the subscales, and the association between FC values of the two ROIs in the motivation network were calculated using CONN with age and gender as covariates of a functional connectivity analysis. Relationships between GPIUS2 scores and the FC values indicated statistical significance if false discovery rate (FDR) corrected *p*-values were less than 0.05.

A one-sample Kolmogorov–Smirnov test revealed that the data were mixed in their distribution. Therefore, to test the correlations between GPIUS2 and AQ or BDI-II, Pearson’s correlation coefficients were used if an initial exploration of the dataset suggested normal distribution of the data and Spearman’s rank-correlation coefficients were used if the data were not normally distributed. Owing to the exploratory nature of this study, correction for multiple comparisons was not applied for these correlations, and an uncorrected *p*-value of 0.05 was regarded as the statistical threshold of significance.

Mediation analysis was used to investigate whether depressive state (indexed by BDI-II) or autistic traits (indexed by AQ) mediated the association between IU and FC values. A non-parametric bootstrap method was applied to test the mediation path (indirect effect of independent variables on the dependent variable through a mediator). An estimate of the indirect effect was the mean, computed using 2000 bootstrap samples, and the 95% bias-corrected confidence interval. The mediation effect was considered to be significant at *p* < 0.05.

## Results

### Psychological Data

Demographic information and scores on the GPIUS2, BDI-II, and AQ are summarized in Table 1. The scores on the BDI-II and AQ were at subclinical levels (mean [SD] = 6.1 [5.9]/18.5 [7.8], respectively). Although the GPIUS2 has no cutoff value for diagnosing EIU, the mean GPIUS2 total score and the means of the subscales in this study were similar to those from another study of healthy volunteers (Caplan, 2010), suggesting they were also subclinical in level (36.2 [15.7]) (Table 1).

### Correlational Analyses Between GPIUS2 and FC

**FIGURE 1:**
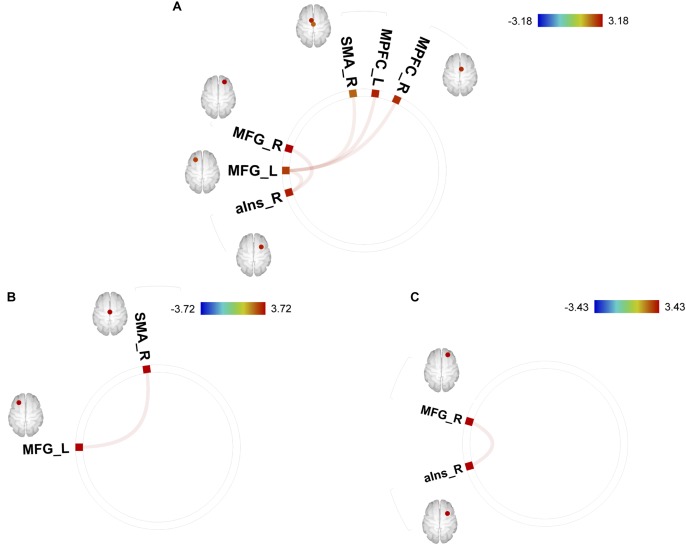
Correlations between **(A)** GPIUS2 total scores/ **(B)** mood regulation subscale/ **(C)** deficient self-regulation and functional connectivity values among motivation network. MFG, middle frontal gyrus; SMA, supplementary motor area; aIns, anterior insula; MPFC, medial prefrontal cortex; L, left; R, right; ROIs_Motivation. Mo, regions of interest within motivation network.

Correlations between GPIUS2 scores and FC within the motivation network are shown in Figure 1. GPIUS2 total scores were positively correlated with FC values between (a) left MFG and bilateral MPFC (left/right; T [115] = 3.07/2.97, *p* = 0.038/0.038), right SMA (T [115] = 2.75, *p* = 0.048), and right aIns (T [115] = 2.65, *p* = 0.048) (b) right MFG and right aIns (T [115] = 3.18, *p* = 0.039). Scores on the GPIUS2 MR subscale were positively correlated with FC between left MFG and right SMA (T [115] = 3.43, *p* = 0.018). Scores on the GPIUS2 DSR subscale were positively correlated with FC between right MFG and right aIns (T [115] = 3.72, *p* = 0.006) (all statistical thresholds were FDR < 0.05). No other FC exhibited significant associations with any GPIUS2 score.

### Mediation Analysis

We found no correlation between BDI-II scores and GPIUS2 total scores or subscale scores (Table 2). BDI-II had no mediating effect on the association between GPIUS2 scores and FC within the motivation network.

**Table 2:** Correlations between BDI-II and GPIUS2 scores.

		*ρ*	*p*-value
GPIUS2_total score		0.148	0.107
GPIUS2_preference for online communication		0.062	0.502
GPIUS2_mood regulation		0.088	0.341
GPIUS2_self regulation		0.162	0.063
GPIUS2_negative outcomes		0.094	0.309


*GPIUS2, Generalized Problematic Internet Use Scale 2; BDI-II, Beck Depression Inventory-II.*

**FIGURE 2:**
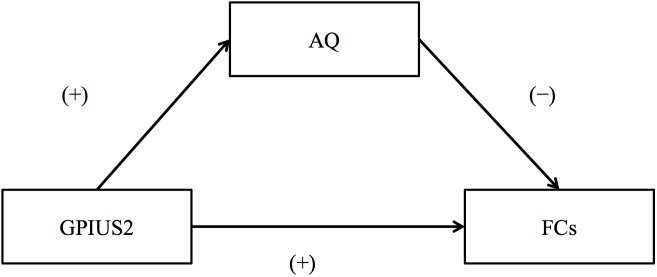
Schematic representation of the relationship among internet use, autistic traits, and functional connectivity. (+) and (–) mean the direction of standardized coefficients between GPIUS2 and FC, GPIUS2 and AQ, and AQ and FC, respectively. GPIUS2, Generalized Problematic Internet Use Scale 2; AQ, Autism Spectrum Quotient; FC, functional connectivity correlated with GPIUS2 scores.

A schematic representation of the relationship among GPIUS2, AQ, and FC is shown in Figure 2. In brief, GPIUS2 scores were positively correlated with AQ scores, regardless of whether total or subscale scores were examined, whereas AQ scores were negatively correlated with FC values (Figure 2). The summary of the mediation analysis is as follows: (1) AQ total scores mediated the association between GPIUS2 total scores and FC between left MFG and right MPFC; (2) The scores on the AQ subscale “Attention switching” mediated the association between GPIUS2 total scores and the FC values of (a) left MFG-MPFC bilaterally, and right SMA; (3) Scores on the “Attention switching” subscale also mediated the association between scores on the GPIUS2 “mood regulation” subscale and FC between the left MFG and right SMA. In the analyses of (1), (2), and (3) mentioned above, the bootstrap method revealed that zero was not within the 95% CI of the indirect effect for GPIUS2 scores (independent variable) on FC values (dependent variable) through AQ scores (mediator), indicating a significant mediation effect (*p* < 0.05, Table 3). No other mediation effect was found on the association between GPIUS2 scores and FC values.

**Table 3:** Results from mediation analysis.

	Effect of independent variable to mediator				Direct effect of mediator				Direct effect of independent variable				Indirect effect of independent variable			
				
	β	*SE*	Standardized regression weight		β	*SE*	Standardized regression weight		β	*SE*	Standardized regression weight		β	*SE*	Boot 95%CL	
	**GPIUS2_total-AQ_total**				**AQ_total-FCs**				**GPIUS2_total- FCs**							
						
MFG_L- MPFC_R	0.161	0.043	0.320	^∗∗^	-0.007	0.002	-0.310	^∗∗^	0.002	0.001	0.206^a^	^∗^	-0.001	0.001	[-0.003378, -0.000021]	^∗^
									0.004	0.001	0.306^b^	^∗∗^				

	**GPIUS2_total-AQ_switch**				**AQ_switch-FCs**				**GPIUS2_total- FCs**							
						
MFG_L- MPFC_L	0.051	0.001	0.361	^∗∗^	-0.018	0.008	-0.209	^∗^	0.003	0.001	0.242^a^	^∗^	-0.001	0.001	[-0.002534, -0.000067]	^∗^
									0.004	0.001	0.317^b^	^∗∗^				
MFG_L- MPFC_R	0.051	0.001	0.361	^∗∗^	-0.034	0.008	-0.400	^∗∗^	0.002	0.001	0.206^a^	^∗^	-0.002	0.001	[-0.003846, -0.000662]	^∗∗^
									0.004	0.001	0.350^b^	^∗∗^				
MFG_L- SMA_R	0.051	0.001	0.361	^∗∗^	-0.018	0.001	-0.251	^∗∗^	0.002	0.001	0.186^a^	^∗^	-0.001	0.001	[-.002457, -0.000110]	^∗^
									0.003	0.001	0.277^b^	^∗∗^				

	**GPIUS2_mood-AQ_switch**				**AQ_switch-FCs**				**GPIUS2_mood- FCs**							
						
MFG_L- SMA_R	0.124	0.038	0.284	^∗∗^	-0.018	0.007	-0.241	^∗∗^	0.008	0.003	0.247^a^	^∗^	-0.002	0.001	[-0.006538, -0.000308]	^∗^
									0.010	0.001	0.315^b^	^∗∗^				


*L, left; R, right; MFG, middle frontal gyrus; MPPC, medial prefrontal cortex; SMA, supplementary motor area; GPIUS2_mood, “Mood Regulation” subscale from the Generalized Problematic Internet Use Scale 2; AQ_switch, “Attention Switching” subscale from the Autism Spectrum Quotient. ^*a*^Without mediating effects, ^*b*^with mediating effects. ^∗^*p* < 0.05, ^∗∗^*p* < 0.01.*

Among the 119 participants, four had high AQ scores (>33 points, which is the cutoff value for suspecting a clinical disorder) and two had high BDI-II scores (>20, suspected to be moderately depressed). One participant had high AQ and BDI-II scores. Three participants had high GPIUS2 total scores (outlier, ≥2 SD from the mean, although no cutoff value exists for the GPIUS2), and among these three, all had either high AQ or BDI-II scores. Therefore, we performed an additional mediation that excluded these five participants (*N* = 114) to test whether including them was valid. These new results were essentially the same as the initial results that were obtained before exclusion.

## Discussion

This is the first study to investigate the neural underpinnings of IU in healthy participants, focusing on the association between IU and the motivation network. We also investigated whether depressive states and autistic traits mediated the relationships between IU and FC values within the motivation network.

Importantly, the current results revealed a positive correlation between IU and FC values within the reward/motivation network. In past EIU studies, evidence suggested that the degree of IU is associated with various neurobiological measures. For example, reports indicate that the severity of IU is associated with lower D_2_ receptor binding potential (Kim et al., 2011; Tian et al., 2014), lower neural activation during anticipation of small and large monetary reward in ventral striatum (Hahn et al., 2014), and reduced RSFC between VTA-NAcc and OFC (Wang R. et al., 2018). These findings are all in line with the concept of attenuated reward-system function. Our current finding of a positive correlation between IU and FC within the motivation network indicates that subclinical levels of IU might act to maintain reward/motivation-network integrity.

Among the reward/motivation network ROIs that we studied, MFG, MPFC, SMA, and aIns were the regions among which FC values between two regions were positively correlated with the degree of IU. Considering that the frontal cortex receives DA projection A10 from the VTA (Fujiwara et al., 2012) and synchronizes with aIns within the reward/motivation network (Kinnison et al., 2012), greater IU in healthy individuals appears to be associated with enhanced reward/motivation-network function among these regions. Although the brain parameters investigated in previous neuroimaging studies of EIU were varied, most (but not all) of them suggest weakened or disturbed function within or connected to regions of the reward/motivation system. Jin et al. (2016) performed multi-modal imaging and reported cortical gray matter volume reduction in the SMA of individuals with EIU. Using SMA as a seed region, they demonstrated lower FC between the SMA and the Ins. Wang R. et al. (2018) focused on investigating FC in the reward system and found reduced RSFC between mesolimbic and cortical regions, supporting the RDS hypothesis for EIU.

The assumption that IU will gradually develop into EIU over a continuum is reasonable. However, our finding of a positive correlation (not negative) between IU and FC might be related to the characteristics of our participants, namely, subclinical levels of IU if any, and without psychiatric comorbidities. Additionally, in most previous studies, participants were pathologically heavy users of online gaming. Therefore, the discrepancy between our current results and those from previous reports might be related to differences between light and heavy Internet users and/or differences in the reasons they used the internet, as most previous studies focused on internet gaming.

Among the GPIUS2 subscales, we found that Mood Regulation (MR) and Deficient Self-Regulation (DSR) were associated with FC within the motivation network. Using the internet for mood regulation has been suggested previously (Caplan, 2002, 2007; LaRose et al., 2003). Caplan (2002) found that MR was a significant cognitive predictor of negative outcomes associated with IU. In a later study, Caplan (2007) argued that socially anxious individuals may prefer online interaction (that is, POSI) because it represents a way to mitigate their anxiety about self-presentation in interpersonal situations. LaRose and colleagues’ work on the socio-cognitive model of unregulated IU also emphasizes the role of MR in the development of DSR (LaRose et al., 2003). DSR refers to a failure to adequately monitor one’s use, judge one’s usage behaviors, and adjust one’s pattern of use (Bandura, 1991). Thus, DSR conceptually represents a higher-order construct that reflects the interplay between compulsive behavior (corresponding to CU) and symptoms of obsessive thought (corresponding to CP) in GPIUS2 (Caplan, 2010). Intuitively, higher scores on the MR and DSR subscales would be expected to lead to negative outcomes in daily behavior and mental health. However, as long as their scores do not reach pathological levels, as we found here, higher scores on these subscales might be expected to have positive effects that encourage individuals to engage in various activities through the internet, which might increase their general levels of motivation. In contrast, the other two subscales (POSI and “Negative Outcomes”) were not associated with FC within the reward/motivation network. One possible reason is that a floor effect emerged due to the smaller mean scores and smaller variances of these two subscales.

We also asked whether depression or autistic traits mediate the association between IU and FC. Unlike other studies that have suggested significant relationships between EIU and depression (Cheng et al., 2018; Kim et al., 2018; Kitazawa et al., 2018), we did not find any correlations between BDI-II and GPIUS2 scores in the current study. Additionally, our results indicated that depression levels do not mediate (positively or negatively) the relationship between IU and FC. This discrepancy with past results could be due to the low levels of depression in the current study sample.

In contrast, AQ total score and the “Attention switching” subscale did mediate the association between IU and FC values. GPIUS2 scores were positively correlated with AQ scores (Table 3, “Effect of independent variable to mediator”), regardless of whether total or subscale scores were analyzed. AQ scores were negatively correlated with FC (Table 3, “Direct effect of mediator,” Figure 2). Importantly, correlation coefficients between IU and FC values increased after controlling for autistic traits, indicating that autistic traits at subclinical levels might have attenuated the positive effects of IU on the integrity of the motivation network. Indeed, autistic traits in non-clinical populations have been suggested to be associated with EIU (Finkenauer et al., 2012; Romano et al., 2013, 2014; Liu S. et al., 2017; So et al., 2017). Thus, non-clinical levels of IU appear to share common factors with EIU, in the sense that autistic traits negatively impact the ability to maintain and foster motivation in both situations. Because the internet is an important and growing source of information and of connectedness with others, and because it might help individuals with autistic traits by offering a safe environment in which to interact with others (Burke et al., 2010), approaches to encourage safe use of the internet should be considered. Previous research regarding IU has reported that autistic traits in the general population were associated with compulsive IU (Finkenauer et al., 2012). Other studies have reported that obsessive traits in particular (i.e., restricted and/or repetitive tendencies) were associated with EIU in clinical and non-clinical populations with greater autistic traits (MacMullin et al., 2016; Shane-Simpson et al., 2016). Lower social connectedness has been reported to be associated with EIU in individuals with non-clinical (but higher) autistic traits (Liu S. et al., 2017). Taken together, these findings suggest that exacerbation of “restricted and/or repetitive tendencies” might lead to compulsive use of the internet in situations in which individuals are socially isolated. If so, IU while maintaining various offline activities might prevent EIU. In addition, given the effect of gender on EIU characteristics (Finkenauer et al., 2012) and the relationship between EIU and decreased emotional regulation and trait-anxiety in individuals with higher autistic traits (Romano et al., 2014; Liu S. et al., 2017), individual factors should be considered when developing interventions for EIU.

The current study has several limitations that should be considered. First, we did not investigate the relationship between IU and FC in participants with a clinical disorder. Determining appropriate levels of IU in terms of mental health promotion will require further investigation of clinical cases and comparison with healthy volunteers. Second, the current study had a cross-sectional design. A longitudinal follow-up study is necessary to clarify the causal relationship between IU and FC. Finally, we did not examine the details of participant internet use, such as its purpose (e.g., internet browsing, shopping, gaming, or SNS), or the devices each participant used on a daily basis (e.g., PC vs. mobile device). Future studies are needed to determine whether the purpose or type of device used for IU have an effect on brain functional connectivity within the reward/motivation system.

## Conclusion

We examined the neural underpinnings of IU in healthy participants and found a positive effect of IU on brain function and mental health. However, the results also revealed that the presence of autistic traits changed this relationship. A longitudinal follow-up study investigating both clinical (internet-addicted individuals) and subclinical populations is needed to clarify appropriate levels of IU.

## Author Contributions

HF conceived, designed, and conducted the experiments, acquired and analyzed the data, and drafted the manuscript. TM, SY, and HF contributed to the conception of the study, interpretation of data, and revisions for critically important intellectual content. KK, TU, and NO contributed to the design and data acquisition, interpretation of data, and drafting the manuscript. All authors approved the final manuscript for submission and agree to be accountable for all aspects of the work, including the assurance that questions related to the accuracy or integrity of any part are appropriately investigated and resolved.

## Conflict of Interest Statement

The authors declare that the research was conducted in the absence of any commercial or financial relationships that could be construed as a potential conflict of interest.

## Footnotes

1 http://www.fmrib.ox.ac.uk/fsl

2 www.nitrc.org/projects/conn

3 http://www.fil.ion.ucl.ac.uk/spm
